# Temporospatial Analysis and New Players in the Immunology of Amyotrophic Lateral Sclerosis

**DOI:** 10.3390/ijms19020631

**Published:** 2018-02-23

**Authors:** Abhirami K. Iyer, Kathryn J. Jones, Virginia M. Sanders, Chandler L. Walker

**Affiliations:** 1 Anatomy and Cell Biology Department, Indiana University School of Medicine, Indianapolis, IN 46202, USA; abhiiyer@iupui.edu (A.K.I.); kjjones1@iupui.edu (K.J.J.); 2 Richard L. Roudebush Veterans Affairs Medical Center, Indianapolis, IN 46202, USA; 3 Department of Cancer Biology and Genetics, The Ohio State University Wexner Medical Center, Columbus, OH 43210, USA; sanders.302@osu.edu; 4 Department of Biomedical and Applied Sciences, Indiana University School of Dentistry, Indianapolis, IN 46202, USA

**Keywords:** amyotrophic lateral sclerosis (ALS), immune response, neuroimmunology, motor neuron disease

## Abstract

Amyotrophic lateral sclerosis (ALS) is a neurodegenerative disorder characterized by progressive loss of lower and upper motor neurons (MN) leading to muscle weakness, paralysis and eventually death. Although a highly varied etiology results in ALS, it broadly manifests itself as sporadic and familial forms that have evident similarities in clinical symptoms and disease progression. There is a tremendous amount of knowledge on molecular mechanisms leading to loss of MNs and neuromuscular junctions (NMJ) as major determinants of disease onset, severity and progression in ALS. Specifically, two main opposing hypotheses, the dying forward and dying back phenomena, exist to account for NMJ denervation. The former hypothesis proposes that the earliest degeneration occurs at the central MNs and proceeds to the NMJ, whereas in the latter, the peripheral NMJ is the site of precipitating degeneration progressing backwards to the MN cell body. A large body of literature strongly indicates a role for the immune system in disease onset and progression via regulatory involvement at the level of both the central and peripheral nervous systems (CNS and PNS). In this review, we discuss the earliest reported immune responses with an emphasis on newly identified immune players in mutant superoxide dismutase 1 (mSOD1) transgenic mice, the gold standard mouse model for ALS.

## 1. Amyotrophic Lateral Sclerosis (ALS)

Motor neuron diseases are devastating and encompass a variety of disorders that lead to death of motor neurons and resulting loss of motor function, dysphagia and respiratory failure. Of these diseases, amyotrophic lateral sclerosis (ALS) is the most common. ALS frequently afflicts adults in middle age (40–60 years old) with an average lifespan of 3–5 years following diagnosis [1]. Most cases of ALS are sporadic in nature, while approximately 20% of familial forms of ALS (FALS) are caused by mutations in the gene for copper/zinc superoxide dismutase (SOD1). Clinically, ALS treatment comprises of riluzole which extends lifespan moderately and edaravone, a free radical scavenger approved for clinical use recently in 2017. Although promising, edaravone confers neuroprotection only when started during early stage of the disease with its long term clinical efficacy yet to be established [2]. Despite the disadvantages of using rodent models of ALS overexpressing mSOD1, it is the most widely used model and closely mimics the pathogenic progression observed in human ALS [3].

There is currently a delay of more than one year between symptom onset and clinical diagnosis, likely due to the underestimation of initial symptoms by patients and clinicians as they are similar to those resulting from various etiologies [4]. ALS is considered a diagnosis based on exclusion of other possible causes. This delay represents a lost temporal opportunity for initiating therapies in a timely manner to treat ALS. Before symptoms even begin, peripheral disconnection of motor axons from target musculature is documented to take place in both ALS patients and mouse models of the disease [5]. As there are currently no effective biomarkers for ALS, the delay between peripheral disease initiation and symptom onset represents another lost therapeutic window of opportunity. These issues underscore the critical need for better understanding of risk factors, initiators of disease, and biomarkers for early identification of individuals at elevated risk for ALS development and progression.

Research into the immune system influence in ALS has yielded important clues on possible markers that could be targeted to tackle the disease before it has progressed too far. Much research into immune system involvement in different stages of ALS disease has been covered to date [6,7]. However, recent research in ALS patients and animal models has identified a role for new immune system components in disease onset and progression. This review will discuss these new findings in the context of prior research and lay emphasis on the temporal and spatial effects of the observed immune activation in ALS.

## 2. New Immune Players in ALS

In addition to MN cell intrinsic molecular defects, secondary mechanisms including toxic glial microenvironment, immune system influence on glial activation and neuromuscular junction (NMJ) maintenance, all converge to modify the disease course in ALS [8]. Here, we discuss the impact of peripheral immune cell interactions with CNS resident glia on MNs and shed light on newly identified interactions between immune system and each component of the NMJ.

### 2.1. Peripheral Immune System—CNS Glia Interactions

Selective reduction of mSOD1 expression in microglia or astrocytes significantly extends the lifespan of ALS mice, thereby establishing a role for glial cells in MN degeneration [9,10]. Glial reactivity, hypertrophy and changes in inflammatory marker expression have been extensively demonstrated in the mSOD1 model and post-mortem tissue of ALS patients [11]. There is also a growing body of work suggesting peripheral immune system involvement in balancing glial neurotrophic and neurotoxic functions in ALS. Accelerated disease progression is observed in mSOD1 mice crossed with T cell receptor β chain (TCRβ) deficient mice that lack αβ+ CD4+ and CD8+ T cells relative to mSOD1 mice [12]. In this study, T cell deficiency leads to decreased microglial reactivity with no effect on astrocytes. Thus, lack of T cells ablates a neuroprotective pathway involving reduced production of neurotrophic factor, IGF-1, in mSOD1 microglia. When mSOD1 mice are bred to a *CD4*^−/−^ background that lack CD4+ T cells, a similar significant reduction in their lifespan is observed relative to mSOD1 mice. The mSOD1 *CD4*^−/−^ mice also exhibit attenuated microglial and astrocyte reactivity. Lack of CD4+ T cells alters the glia phenotype towards toxicity, owing to reduced expression of neurotrophic factors such as IGF-1, GDNF, BDNF; anti-inflammatory cytokines such as IL-4, TGF-β and increased expression of proinflammatory molecules such as TNF-α, NOX2 in the lumbar spinal cords of these mice [13]. In a more direct approach, this group also showed that passive transfer of wild-type (WT) CD4+ T cells, or subpopulation of CD4+ T cells, namely, regulatory T cells (Tregs) from stable disease phase mSOD1 mice leads to neuroprotection in mSOD1/*RAG2^−/−^* recipient mice. The authors attribute the CD4+ T cell-mediated neuroprotection to the presence of IL-4+ Tregs, which in turn promote anti-inflammatory M2-like microglia phenotype and their production of neurotrophic factors. Extending these observations to human ALS patients, this study also reported decreased numbers of Tregs in blood to correlate with faster disease progression. Contrary to this, Tregs isolated from mSOD1 mice during the late rapid progressive phase and transferred into mSOD1/*RAG2^−/−^* recipients fails to prolong disease duration and extend survival [14]. Tregs, a distinct CD4+ T cell subpopulation involved in self-tolerance and immune homeostasis, have well documented suppressive effects on innate and adaptive immune cells [15]. The results from these studies suggest that the ability of the early disease phase Tregs to sustain M2-like microglial responses is lost with disease progression leading to a predominant M1-like microglial phenotype at end stage.

The T helper 2 (Th2) sub-population of CD4+ T cells have also been shown to be crucial in sustaining MN survival after facial nerve axotomy (FNA), a peripheral nerve injury model [16]. Reconstitution of immunodeficient RAG2−/− mice with whole splenocytes or CD4+ T cells from WT or mSOD1 donor mice leads to differences in the CNS molecular responses to FNA. Specifically, RAG2−/− recipients supplemented with mSOD1 whole splenocytes show increased astrocyte activation and neuronal cell death pathway triggers such as Fas and nNOS, potentially the underlying cause of reduced MN survival in this group after FNA [17]. Taken together, these studies suggest that peripheral immune cells, particularly CD4+ T cells modulate CNS neuroprotective pathways via a direct impact on glial responses to disease or injury. On the other hand, a newer study in mSOD1 mice selectively overexpressing TGF-β in astrocytes shows reduced CD4+ T cell infiltration and their increased polarization towards proinflammatory IFN-γ+ Th1 over IL-4 producing Th2 phenotype in the spinal cord. Further, there was significant decrease in microglial immune activation markers and IGF-1 expression in the lumbar spinal cord of these mice [18]. This study proposes higher astrocytic TGF-β1 as a negative prognostic factor in mSOD1 mice owing to its inhibition of microglia and CD4+ T cell-mediated neuroprotective inflammation. Although neuroprotection conferred by CD4+ T cells is well established, the nature and source of antigens (Ags) and the compartment (CNS or PNS) where these lymphocytes are activated in ALS are currently unclear.

Another recent study discovered a role for MN-specific major histocompatibility complex (MHC) class I molecules, that classically trigger CD8+ T cell based adaptive immune responses, in protection from astrocyte-induced toxicity. MHC class I expression was highly localized to motor axons over cell bodies and was nearly absent in spinal cords of end stage sporadic and familial ALS patients in comparison to healthy controls. Furthermore, in contrast to WT, mSOD1 astrocytes when co-cultured with MNs induces downregulation of MHC class I expression. Conditioned media from mSOD1 astrocyte cultures alone is sufficient to cause this downregulation via induction of endoplasmic reticulum (ER) stress pathways in MNs. Overexpression of MHC class I in MNs rescues them from the astrocyte-induced toxicity, delays disease progression and enhances survival in mSOD1 mice [19].

**Figure 1:**
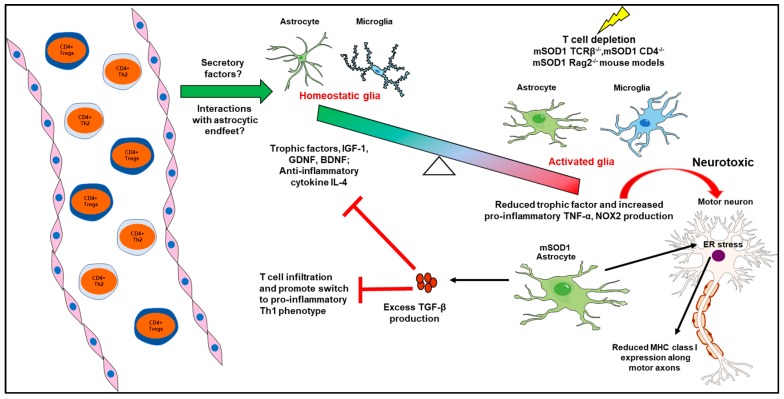
CD4+ T cells tip the balance between glial neurotrophism and neurotoxicity. Circulating CD4+ T cells (specifically, Tregs and Th2 cells) via yet-to-be identified mechanisms promote microglial and astrocyte production of trophic factors and anti-inflammatory cytokines. Depletion of CD4+ T cells in mSOD1 mice via different genetic approaches switches them to an activated proinflammatory phenotype with neurotoxic properties. BDNF: brain-derived neurotrophic factor; ER: endoplasmic reticulum; GDNF: glial cell-derived neurotrophic factor; ; IGF-1: insulin-like growth factor 1; IL-4: interleukin-4; MHC: major histocompatibility complex class I; mSOD1:mutant SOD1; NOX2: NADPH oxidase isoform 2; Tregs: regulatory T cells ; Th2: T helper 2; TCRβ: T cell receptor β; TGF-β: transforming growth factor-β; TNF-α: tumor necrosis factor-α.

Overall, as summarized in Figure 1, glia-peripheral immune interactions have a strong influence on neuronal health that during the early stages of ALS disease strive to sustain MN survival while at the end stage, due to a vicious circle of excessive inflammation, become the pathological basis for neurodegeneration.

### 2.2. Immune System Interactions with the Tripartite Neuromuscular Junction

**Figure 2:**
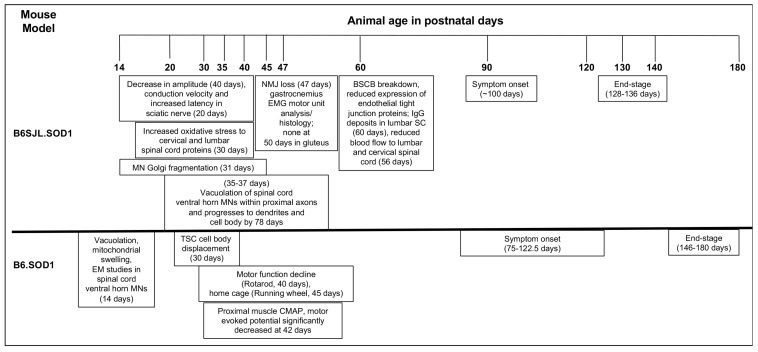
A timeline of reported molecular, histological and functional events in the lifetime of mSOD1^G93A^ mice.

A neuromuscular junction (NMJ), serving as the functional units for skeletal muscles, are comprised of presynaptic MN axon terminals, the post-synaptic skeletal muscle and the synapse associated glial cells, Schwann cells (SC; also referred to as perisynaptic or terminal Schwann cells, TSC). Dynamic communication between all three components contributes to formation, maturation and maintenance of NMJs; any perturbations in this crosstalk results in NMJ instability, axon retraction and eventually muscular atrophy [20]. In fact, denervation in motor end plates is reported in mSOD1 mice as early as postnatal day (PND) 47, supporting the idea of distal axonal pathology preceding neuronal degeneration and symptom onset in ALS [5] (Figure 2).

Research on CNS synaptic pruning led to the surprise identification of immune system components in mediating synapse elimination, particularly, during development. Examples of such molecules include the MHC class I proteins, associated accessory protein β2m and complement cascade proteins, immune molecules that are typically involved in pathogen elimination [21,22]. In contrast to prevalent dogma on lack of MHC expression in the CNS and the immune privilege to escape T cell recognition, reports on mRNA expression for MHC class I and β2m is response to virus infections or nerve injury dates back more than two decades [23]. For example, MHC class I is upregulated in rat facial MNs and skeletal muscle in response to FNA. Using a nerve cut vs crush injury, MHC class I is implicated to play a role in regeneration in this study [24]. Almost 20 years later came the first evidence for subcellular localization of MHC class I molecules in MN axons and their presynaptic terminals at the NMJ. The expression is strongly upregulated after sciatic nerve axotomy with its absence leading to fewer TSCs covering the NMJ, abnormalities during regeneration and delayed recovery of motor function. This study also put forth a non-immune function for MHC class I in axonal regeneration. Further studies carried out to identify putative MHC class I receptors identified cognate PIR-B receptor, associated with synaptic plasticity in the CNS and on SCs in the NMJ [25].

Several newer studies in the past 5 years have investigated the potential role of MHC class I in ALS neurodegeneration. β2m is an essential component that noncovalently associates with MHC class I and is also expressed in MNs, their axons and promote recovery after nerve axotomy or crush injuries. Removing β2m from mSOD1 mice considerably shortens disease duration and survival signifying a positive role for this molecule in regeneration and the disease process [26]. In a rather elegant study, Nardo et al, compared and contrasted components of the immune system in the NMJs of slow and fast progressing mSOD1 mice [27]. Indeed, upregulation of immune molecules including MHC class I and infiltration of peripheral immune CD8+ T cells and macrophages are seen along motor axons and the NMJ of slow progressing mice but lacking in the fast progressing mice [27]. These data strongly suggest that activating the immune response around the peripheral nervous system is crucial to delaying denervation and thereby control disease progression in mSOD1 mice.

### 2.3. Schwann Cell (SC) Abnormalities and Immune Interactions

Several studies in other areas of neuroscience research have identified key roles for immune and inflammatory responses in pathology or disruption of normal SC function in peripheral nerve. Intriguingly, SCs appear to demonstrate many characteristics that directly or indirectly influence immune interactivity with the peripheral immune system in normal and disease conditions. SCs can exhibit properties of an immune cell including Ag recognition, presentation, altering local immune response and termination [28]. T cells exhibit heightened activation in recognition of peptide Ags in the context of MHC molecules together with co-stimulatory signals from the Ag presenting cell. In inflammatory nervous system disorders, Schwann cells increase expression of a co-stimulatory molecule, BB-1, through their maturation toward a myelinating phenotype, though constitutive expression on immature or non-myelinating SCs could be involved in demyelinating disorders [29]. During NMJ dismantling, axonal retraction and demyelination in ALS, several antigens are likely presented by SCs that trigger immune responses to promote debris clearance and repair, but also exacerbate disease progression. Immune involvement in neuroinflammatory conditions could catalyze physiological changes in the TSC for the initiation of NMJ degeneration and continue throughout the entire peripheral nerve breakdown process. 

In many ways, the responses seen in progressive peripheral nerve pathology is similar between aged and transgenic ALS animal models. Neuromuscular alterations and disconnections occur, and glial and neuronal physiology are modified over time. As animals age, research suggests down-regulation of immune and cytoregulatory functions occur including diminished macrophage infiltration and SC involvement in myelin debris clearance following nerve injury and repair [30]. As SCs can recruit macrophages via CCL2 release [31], and this release is reduced over time in the aged nerve, it may be that decreased ability of SCs to recruit macrophages plays a causative role in this observed response. However, macrophage phagocytic functions also decrease with increased age, thus, a combination of factors are likely to contribute to these phenomena.

The study of SCs and their potential role in ALS is not new [32,33,34,35,36,37], however, recent studies have begun linking neuroinflammatory and immunologic responses to peripheral nerve myelination and SC function in the disease [27,38]. In addition to MN axons, MHC class I expression is elevated in sciatic nerve SCs of a C57SOD1^G93A^ mouse model of ALS and coincides with slower disease progression compared to faster progressing 129SvSOD1^G93A^ model despite expressing the same amount of mSOD1 transgene [27]. The implications of this finding are that there is enhanced MHC class I recruitment of CD8+ T cells to the peripheral nerve in the C57SOD1^G93A^ model and the sequela of yet to be recognized immunologic events involving SCs impacts disease progression and severity. The SCs identified to show increased MHC class I expression were not located at the NMJ, but rather within the nerve itself where the cells closely interact with peripheral axons. This evidence is also interesting, as CD8+ T cells are often associated with cytotoxicity, though their response in this study is associated with less degeneration and slower disease progression.

In contrast to these findings, a recent study identified a correlation between increased mast cell infiltration at the extensor digitorum longus (EDL) muscle NMJs and increased paralysis progression in mSOD1^G93A^ mice [38,39]. This increase in mast cells at the NMJ temporally corresponds with the loss of TSC coverage and presence at the NMJ in the SOD1 rat. The evidence presented by Trias et al. [38] suggests mast cells play a role in the NMJ dismantling including TSC retraction as masitinib, a c-kit tyrosine kinase inhibitor used for mast cell tumors and in neuroinflammatory diseases, diminished this breakdown of the NMJ in the SOD1 rat in the post-paralysis stage. Based on these findings, the functional status of the mast cell is important in its role in NMJ pathology as observed in this experimental model of ALS.

Though ALS is a MN disease, new evidence suggests the SC expression of MHC class II in a rodent nerve injury model could contribute to sensory hyperalgesia in these animals [40]. Deletion of MHC class II in myelinating SCs reduced this hyperalgesia, which supports a more complicated role for Schwann cells in peripheral nerve function in disease or injury conditions. Since peripheral nerves are often mixed motor and sensory nerves, any effects of motor component dysfunction on sensation is likely mediated by changes in SC physiology including MHC class II-mediated Ag presentation. Such a response could at least partially explain the influx of adaptive immune T cells in degenerating peripheral nerve in ALS. However, this finding is new and whether Schwann cells present Ags to T cells via MHC class II requires additional study in ALS. Still, the Ag-presentation capacity of SCs raises many possibilities concerning the neuroimmune involvement of these peripheral myelinating glia in ALS. Table 1 provides a temporal overview of research related to tissue-specific immune interaction with neuromuscular components in ALS. 

### 2.4. Skeletal Muscle Immune System Cross-Talk

Progressive weakness and atrophy in skeletal muscles is the cardinal feature of ALS. Skeletal muscles that are targets of lower MNs, also provide cues for different aspects of NMJ development and structural maintenance [41]. Thus, in addition to undergoing denervation induced atrophy, skeletal muscle might also play an active role in NMJ dismantling and the ensuing inflammation in ALS. Muscle pathology in mSOD1 mice is evident long before MN cell death in the CNS [42]. Interestingly, motor terminal abnormalities are prominent in mSOD1 fast-twitch muscles as early as post-natal 31–40 days of age, with similar decline in slow-twitch muscles occurring only around onset of disease symptoms [43]. An increased vulnerability of axons innervating fast-twitch muscles to degenerate is also observed in ALS patients [44,45]. mSOD1 expression in only skeletal muscle leads to NMJ abnormalities and muscle pathology largely due to mitochondrial dysfunction and activation of cell death pathways [46]. Several studies show skeletal muscle as the primary target of mSOD1-mediated toxicity [47], however, the jury is still out on whether muscular mSOD1 plays a causal role in MN degeneration.

The role played by peripheral immune cells and molecules on muscle pathology in ALS is only starting to emerge. Muscle cells express classical Ag presenting molecules, MHC class I and II, either constitutively at low levels or after exposure to IFN-γ such as in inflammatory muscle diseases [48], and also express a member of the B7 family of co-stimulatory molecules, ICOSL that is crucial to fine-tune T-cell responses [49]. Whereas axonal MHC class I expression is attributed to neuroprotection in ALS, muscular expression of MHC molecules in inflammatory myopathies is usually associated with lesion and subsequent atrophy [50,51]. Furthermore, muscle biopsy specimens from inflammatory myopathy and degenerative muscle disease patients were found to express a non-classical MHC class I-like molecule called HLA-G [52,53]. HLA-G, a tolerogenic Ag classically studied in maternal-fetal tolerance, interacts with cytotoxic T lymphocytes and natural killer (NK) cells to mediate tissue protection from immune-mediated damage [54,55]. Negative co-stimulatory molecules, B7-H1 and B7-H3, are also markedly increased in muscle fibers from inflammatory myopathies, whereby, via their strong immune-inhibitory properties, they protect skeletal muscle from immune attack in such conditions [56,57]. Expression of MHC and positive or negative co-stimulatory molecules enables muscle cells to act as facultative Ag presenting cells capable of modulating local immune responses, thereby, warranting characterization of skeletal muscle expression of these molecules at different stages of ALS disease.

Stressed or dying cells can serve as a source of endogenous danger signals called damage associated molecular patterns (DAMPs), that can trigger an inflammatory immune response by binding to a family of innate immune sensors called the Toll-like receptors (TLRs), of which there are 10 in humans and 13 in mice. TLR signaling is associated with proinflammatory cytokine production and enhanced Ag presentation to naive T cells, thus it is involved in activation of both innate and adaptive immune responses [58]. TLRs in CNS glia are capable of being activated by mSOD1 itself, whereby such activation has been attributed to glia toxicity on one hand [59,60] and on the other, mSOD1 mice lacking TLR signaling exhibit earlier disease onset and shorter life span [61]. Several findings show expression of multiple TLRs within skeletal muscle [62,63,64]. Interestingly, there are more TLR expressing cells in slow-twitch fibers than fast-twitch muscles in a mouse model of Duchenne muscular dystrophy, a degenerative myopathy with more muscle pathology in fast-twitch muscles like that observed in ALS [65]. However, due to technical challenges, it was difficult to correlate TLR expression levels with the extent of muscle damage in that study. Nonetheless, spatial differences in TLR expression could impact the downstream cytokine/chemokine response and ongoing inflammation. Targeting TLRs as a treatment modality is being actively pursued in ALS [66,67]; therefore, characterizing specific TLR pathways involved in neurotoxicity and muscle pathology will be crucial in recognizing targets for altering local immune responses. Another class of majorly studied innate immune molecules implicated in ALS disease are the complement proteins. Numerous studies have identified components of the complement pathways to be upregulated in the serum, cerebrospinal fluid, spinal cord and motor cortex of ALS patients and mSOD1 mice [68,69,70,71]. In line with the dying back hypothesis, a study shows complement activation products C3/C3b and C1q in motor end plates of mSOD1 mice by postnatal day 47 around the same time that NMJ denervation begins [72,73]. It is possible that similar to elimination of synapses during development [74], complement proteins play a role in NMJ degeneration in ALS. Another recent supports this hypothesis by data showing dysregulation of terminal complement protein C5a-C5aR1 signaling axis within skeletal muscle leading to chronic accumulation of peripheral macrophages that switches them to inflammatory phenotype and exacerbate muscle damage [75].

In response to injury or damage, the skeletal muscle can act as a local source of immune mediators by releasing proinflammatory cytokines (IL-6, IL-8), chemokines (RANTES, MCP-1) and express surface molecules (ICAM-1) that can exert autocrine and paracrine effects allowing recruitment of neutrophils, macrophages and adaptive immune T cells into the damaged muscle [76,77,78]. In addition, studies in acute injury models highlight the importance of muscle resident immune cells including CD8+ cytotoxic T cells, regulatory T (Treg) cells, neutrophils and eosinophils in not just the immediate response to injury but also by producing inflammatory cytokines, IFN-γ and TNF-α, they regulate key transcription factors in myogenic precursor satellite cells involved in repair and regeneration [79]. Muscle resident fibro/adipocyte progenitors (FAPs), bipotent cells capable of giving rise to fibroblasts or adipocytes, are in close association with regenerating muscle fibers and control the balance between muscle regeneration and fatty degeneration. Interestingly, eosinophil-derived, anti-inflammatory cytokines, IL-4 and IL-13, are critical for FAPs to promote muscle regeneration primarily, through blocking their adipogenic differentiation, enhanced proliferation and debris clearance in the injured muscle [80]. Although, the role of eosinophils in ALS has not been directly investigated, elevated levels of eosinophil-derived neurotoxin and RANTES, a chemokine factor for eosinophils in serum and histological evidence in skeletal muscle biopsies from ALS patients is documented [81,82,83]. Also, peripheral nerves can produce eosinophil chemotactic factors and adhesion molecules to promote their recruitment upon which, eosinophils potentially cause axonal damage of MNs via release of neuronal reactive oxygen species and toxic proteins [84,85,86]. Taken together, these studies emphasize the need for a more direct approach such as crossing the eosinophil-deficient ΔdblGATA mice to the mSOD1ALS mice to investigate the role of eosinophils in ALS disease both at the CNS and at the NMJ [87].

Potent suppressors of immune responses, the CD4+ T regulatory cells (Tregs), also work to maintain tissue homeostasis in non-immune organs, including injured and regenerating skeletal muscle. Skeletal muscle Tregs are a phenotypically and functionally distinct population characterized by their signature expression of IL-33 cytokine receptor, ST2 and growth factor amphiregulin (Areg). These cells accumulate around the time when infiltrating myeloid cells switch from proinflammatory to a regenerative phenotype [88]. An interesting study of acute muscle injury in aged mice uncovered an unexpected role for nerve-associated FAPs as IL-33 producers, the ligand for the Treg ST2; with there being reduced IL-33 production in skeletal muscle from aged mice [89]. Thus, this study suggests an important role for the IL-33:ST2 axis for muscle Treg accumulation and regeneration. Sera from ALS patients were found to have significantly reduced levels of IL-33 levels and elevated levels of soluble ST2 receptors relative to healthy controls [90]. In another study, IL-33 released by oligodendrocytes and astrocytes after CNS injury works with yet-to-be identified factors to steer macrophages towards an anti-inflammatory, pro-regenerative M2 phenotype [91]. Targeting IL-33 to improve disease outcome has been promising in other neurodegenerative diseases such as experimental autoimmune encephalomyelitis (EAE) and Alzheimer’s [92,93]; therefore, investigating the IL-33:ST2 axis in skeletal muscle pathology and glial toxicity in ALS might be beneficial. 

Another immunoregulatory cytokine expressed by muscle cells in inflammatory myopathies is IL-15. IL-15 expression by skeletal muscle promotes increased cytotoxicity and effector function of CD8+ T cells leading to disease progression in autoimmune myositis [94]. IL-15 is a crucial cytokine for NK cell development, survival and function; both soluble and membrane-bound IL-15/IL-15Rα complexes increase stability of IL-15 and can effectively activate NK cells even at distant sites via a unique, novel ‘trans-presentation’ mechanism of signal transduction. A drop in NK cell numbers with aging is concurrent with declining levels and stability of skeletal muscle IL-15 in aged mice [95]. In ALS, serum IL-15 levels are elevated although there is no correlation with disease onset or duration [96]. Interestingly, in a recent peripheral blood immunophenotyping study, ALS patients also exhibit elevated NK cell counts that inversely correlates with the ALSFRS-R score [97]. In contrast to the suggested molecular parallels in aging and ALS [98,99], IL-15 and NK cell levels are elevated in ALS opposite to their decline in aging. NK cells are neuroprotective via their production of neurotrophic factors such as BDNF, NT-3 in EAE [100] and play deleterious roles in other neurodegenerative diseases such as MS and Alzheimer’s [101,102]. Thus, discovering the role of IL-15/NK cell axis in CNS and NMJ microenvironments of mSOD1 mice can help develop IL-15/NK-cell based therapies for ALS. Skeletal muscle is a promising target tissue to alter the local and systemic immune response in ALS, due to the tissue abundance, easy accessibility and extensive studies on its immune system interactions in vaccine biology. Although transcriptomics analysis of immune markers in ALS skeletal muscle is emerging [103], direct investigation of skeletal muscle-driven immune responses in ALS are currently limited. A detailed study on skeletal muscle-driven immune responses in mSOD1 mice might result in development of cellular immunotherapies or immune-target based interventions for treating ALS.

## 3. Conclusions

**Figure 3:**
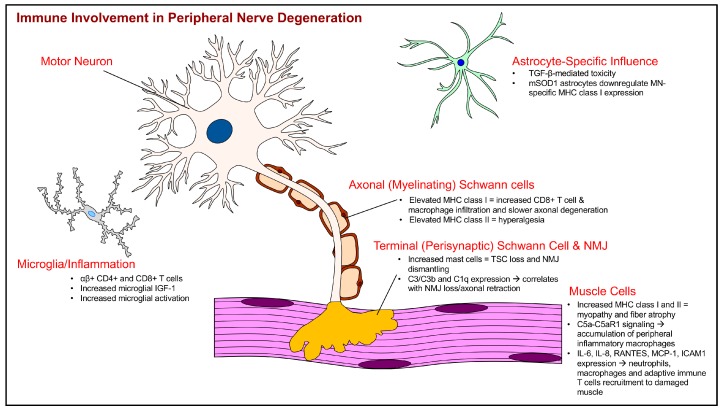
Schematic illustration summarizing peripheral nerve components and immune system involvement in degeneration and pathology.

Research in available animal models has identified key aspects of the temporospatial progression and immune system involvement in neuromuscular breakdown in ALS (Table 1, Figure 2 and Figure 3) [104]. Though such models are not perfect, they have advanced our understanding of complex neuroimmune interactions that also occur in ALS patients. Systemic immune system activation in ALS has also been identified in addition to those at the CNS and PNS, including an increased ratio of neutrophils and monocytes [105], an inflammatory phenotype of circulating dendritic cells [106], and lower Tregs and Foxp3 expression [107]. Such examples highlight the importance of understanding the impact of systemic immune changes in modulating tissue specific disease pathways discussed in the above sections. Further research is necessary to link systemic and localized immune responses in sporadic and familial forms of ALS. Studies thus far on immune-associated therapies for ALS patients have emphasized the non-cell autonomous nature of the disease and the inflammatory component within the CNS microenvironment. Specifically, the inflammatory regulation in the CNS relies heavily on the activation state of astrocytes and microglia and communication with the systemic immune response, and though targeting neuroinflammation with various compounds have worked in animal models, the beneficial results have not translated to humans. Drugs including celecoxib and TNF-α inhibitor thalidomide, [108,109] have not showed significant benefits when used clinically to treat ALS. Immunotherapies including corticosteroids, cyclophosphamide, and cyclosporine have also not shown promising results in clinical treatment of ALS [110,111]. Reasons for this, as summarized by Crisafulli et al. [112] may be due to the delayed time points of delivery during advanced disease stages. One of the problems with many therapies for ALS in humans is that therapy is administered once the disease is diagnosed, and at this point perhaps the disease has progressed beyond the ability to slow or reverse neurodegeneration and subsequent functional decline and paralysis.

Despite our expanding understanding of cellular and physiological contributors to ALS onset and progression, there is still much to learn and identifying such processes and stages in human ALS is difficult. Therefore, improving our ability for early detection of the disease by identifying specific biomarkers that indicate temporospatial state of disease and immunologic and inflammatory correlates with these markers is essential for the best assessment of the efficacy of the variety of clinically-approved and newly developed immune and inflammation-targeted therapies. Also, new animal models that more widely represent the true ALS patient population beyond mutant SOD1 and other hereditary minority subsets of the disease would likely aid in screening such therapies before advancing to human application.

Though recent research has expanded our understanding of the role of the immune response in MN diseases such as ALS, this review highlights not only new findings that contribute to this understanding, but also gaps in knowledge that still remain. ALS is considered a non-cell autonomous disease, as we now know many non-neuronal nervous system cells contribute to pathology, including astrocytes, microglia and neuromuscular interactions. We have focused on highlighting how complex immune system involvement may be in predicting the motor function outcome in this debilitating disease. It will be key for future research to connect findings involving immune involvement in CNS cell responses to those that reside in the peripheral segment: Schwann cells, fibroblasts, muscle cells, etc. Such investigations hold tremendous promise in identifying patterns to characterize immune responses at different disease phases that lead not only to potential therapeutic targets, but also biomarkers to improve speed of diagnosis.

## Figures and Tables

**Table 1:** Temporospatial breakdown of observed immune interactions in ALS.

Reported Immune Cellular/Marker Changes in ALS	ALS Model	Earliest Observed Immune System Changes in Postnatal Days of Age	Reference(s)
Increased lymphocyte cell numbers in spinal cord and altered microglial immune profile	B6SJL.SOD1^G93A^	65 (presymptomatic)	[12]
Reduced growth factors and increased neurotoxic molecular expression in the absence of CD4+ T cells	B6.SOD1^G93A^	~180 (end-stage)	[13]
Increase in percent CD4+CD25+, CD25+FoxP3 Tregs in SOD1 mice blood and reduced Tregs in ALS patients with rapidly progressing disease	B6.SOD1^G93A^ and definite or probable sporadic ALS patients	77 (symptom onset), peak at 112 (stable disease phase) in mice	[14]
Increased β2m in spinal cord of ALS mice	B6.SOD1^G93A^	130 (mid-symptomatic)	[26]
Increase in MHC class I, β2m, LMP7, CCL2, C3, macrophages and CD8+ T cells along axons of peripheral nerve; MHC class I overlap with Schwann cells	B6.SOD1^G93A^	135 (mid-symptomatic)	[27]
Endomysial infiltration of mast cells	SOD1^G93A^ rats NTac:SD-Tg(SOD1^G93A^) L26H	Paralysis onset (187+/−15)	[38,39]
C3 activation products, C1q in motor end plates in mice; C1q and regulators CD55, CD59 on motor end plates in intercostal muscle	B6SJL.SOD1^G93A^; sporadic and familial ALS patients	47 (presymptomatic); post-mortem	[72,73]
C1qB, C4, fB, C3, C5a, and C5aR1 in tibialis anterior (TA) muscles; C5a and its receptor C5aR1 in TA and soleus muscle with C5aR1 localization on macrophages	B6.SOD1^G93A^	Variable between 77 (symptom onset) and 130 (mid-symptomatic) for each listed molecule, however, most molecules are upregulated by 77; at 77 (symptom onset)	[75]
Elevated eosinophil-derived neurotoxin in serum and chemokine RANTES in serum and CSF	ALS patients	Mean ALSFRS-R = 17.8 (SD = 13.29)	[81,82]
Reduced IL-33 and elevated levels of soluble ST2 receptors in serum	ALS patients	met the modified El Escorial criteria for probable or definite ALS	[90]
Elevated serum IL-15 and NK cells	ALS patients	-	[96,97]
Increased macrophage accumulation in sciatic nerve, T cell co-stimulatory pathway upregulated in skeletal muscle, sciatic nerve, spinal cord and in blood samples from ALS patients	B6SJL.SOD1^G93A^ and ALS patients	50 (symptom onset) through 120 (end-stage) in mice	[103]

## References

[1] Talbot K. (2009). Motor neuron disease: The bare essentials. Pract. Neurol..

[2] Petrov D., Mansfield C., Moussy A., Hermine O. (2017). ALS clinical trials review: 20 years of failure. Are we any closer to registering a new treatment?. Front. Aging Neurosci..

[3] Philips T., Rothstein J.D. (2015). Rodent models of amyotrophic lateral sclerosis. Curr. Protoc. Pharmacol..

[4] Kraemer M., Buerger M., Berlit P. (2010). Diagnostic problems and delay of diagnosis in amyotrophic lateral sclerosis. Clin. Neurol. Neurosurg..

[5] Fischer L.R., Culver D.G., Tennant P., Davis A.A., Wang M., Castellano-Sanchez A., Khan J., Polak M.A., Glass J.D. (2004). Amyotrophic lateral sclerosis is a distal axonopathy: Evidence in mice and man. Exp. Neurol..

[6] Puentes F., Topping J., Kuhle J., van der Star B.J., Douiri A., Giovannoni G., Baker D., Amor S., Malaspina A. (2014). Immune reactivity to neurofilament proteins in the clinical staging of amyotrophic lateral sclerosis. J. Neurol. Neurosurg. Psychiatry.

[7] Malaspina A., Puentes F., Amor S. (2015). Disease origin and progression in amyotrophic lateral sclerosis: An immunology perspective. Int. Immunol..

[8] Ilieva H., Polymenidou M., Cleveland D.W. (2009). Non-cell autonomous toxicity in neurodegenerative disorders: ALS and beyond. J. Cell Biol..

[9] Beers D.R., Henkel J.S., Xiao Q., Zhao W., Wang J., Yen A.A., Siklos L., McKercher S.R., Appel S.H. (2006). Wild-type microglia extend survival in pu.1 knockout mice with familial amyotrophic lateral sclerosis. Proc. Natl. Acad. Sci. USA.

[10] Yamanaka K., Chun S.J., Boillee S., Fujimori-Tonou N., Yamashita H., Gutmann D.H., Takahashi R., Misawa H., Cleveland D.W. (2008). Astrocytes as determinants of disease progression in inherited amyotrophic lateral sclerosis. Nat. Neurosci..

[11] Rossi S., Cozzolino M., Carri M.T. (2016). Old versus new mechanisms in the pathogenesis of ALS. Brain Pathol..

[12] Chiu I.M., Chen A., Zheng Y., Kosaras B., Tsiftsoglou S.A., Vartanian T.K., Brown R.H., Carroll M.C. (2008). T lymphocytes potentiate endogenous neuroprotective inflammation in a mouse model of ALS. Proc. Natl. Acad. Sci. USA.

[13] Beers D.R., Henkel J.S., Zhao W., Wang J., Appel S.H. (2008). CD4+ T cells support glial neuroprotection, slow disease progression, and modify glial morphology in an animal model of inherited ALS. Proc. Natl. Acad. Sci. USA.

[14] Beers D.R., Henkel J.S., Zhao W., Wang J., Huang A., Wen S., Liao B., Appel S.H. (2011). Endogenous regulatory T lymphocytes ameliorate amyotrophic lateral sclerosis in mice and correlate with disease progression in patients with amyotrophic lateral sclerosis. Brain.

[15] Rothstein D.M., Camirand G. (2015). New insights into the mechanisms of Treg function. Curr. Opin. Organ Transplant..

[16] Deboy C.A., Xin J., Byram S.C., Serpe C.J., Sanders V.M., Jones K.J. (2006). Immune-mediated neuroprotection of axotomized mouse facial motoneurons is dependent on the IL-4/STAT6 signaling pathway in CD4+ T cells. Exp. Neurol..

[17] Setter D.O., Runge E.M., Schartz N.D., Kennedy F.M., Brown B.L., McMillan K.P., Miller W.M., Shah K.M., Haulcomb M.M., Sanders V.M. (2017). Impact of peripheral immune status on central molecular responses to facial nerve axotomy. Brain Behav. Immun..

[18] Endo F., Komine O., Fujimori-Tonou N., Katsuno M., Jin S., Watanabe S., Sobue G., Dezawa M., Wyss-Coray T., Yamanaka K. (2015). Astrocyte-derived TGF-β1 accelerates disease progression in ALS mice by interfering with the neuroprotective functions of microglia and T cells. Cell Rep..

[19] Song S., Miranda C.J., Braun L., Meyer K., Frakes A.E., Ferraiuolo L., Likhite S., Bevan A.K., Foust K.D., McConnell M.J. (2016). Major histocompatibility complex class I molecules protect motor neurons from astrocyte-induced toxicity in amyotrophic lateral sclerosis. Nat. Med..

[20] Li L., Xiong W.C., Mei L. (2017). Neuromuscular junction formation, aging, and disorders. Annu. Rev. Physiol..

[21] Oliveira A.L., Thams S., Lidman O., Piehl F., Hokfelt T., Karre K., Linda H., Cullheim S. (2004). A role for MHC class I molecules in synaptic plasticity and regeneration of neurons after axotomy. Proc. Natl. Acad. Sci. USA.

[22] Stevens B., Allen N.J., Vazquez L.E., Howell G.R., Christopherson K.S., Nouri N., Micheva K.D., Mehalow A.K., Huberman A.D., Stafford B. (2007). The classical complement cascade mediates CNS synapse elimination. Cell.

[23] Thams S., Oliveira A., Cullheim S. (2008). MHC class I expression and synaptic plasticity after nerve lesion. Brain Res. Rev..

[24] Maehlen J., Nennesmo I., Olsson A.B., Olsson T., Schroder H.D., Kristensson K. (1989). Peripheral nerve injury causes transient expression of MHC class I antigens in rat motor neurons and skeletal muscles. Brain Res..

[25] Thams S., Brodin P., Plantman S., Saxelin R., Karre K., Cullheim S. (2009). Classical major histocompatibility complex class I molecules in motoneurons: New actors at the neuromuscular junction. J. Neurosci..

[26] Staats K.A., Schonefeldt S., Van Rillaer M., Van Hoecke A., Van Damme P., Robberecht W., Liston A., Van Den Bosch L. (2013). Β-2 microglobulin is important for disease progression in a murine model for amyotrophic lateral sclerosis. Front. Cell. Neurosci..

[27] Nardo G., Trolese M.C., de Vito G., Cecchi R., Riva N., Dina G., Heath P.R., Quattrini A., Shaw P.J., Piazza V. (2016). Immune response in peripheral axons delays disease progression in SOD1 G93A mice. J. Neuroinflamm..

[28] Meyer zu Horste G., Hu W., Hartung H.P., Lehmann H.C., Kieseier B.C. (2008). The immunocompetence of schwann cells. Muscle Nerve.

[29] Murata K., Dalakas M.C. (2000). Expression of the co-stimulatory molecule BB-1, the ligands CTLA-4 and CD28 and their mRNAs in chronic inflammatory demyelinating polyneuropathy. Brain.

[30] Painter M.W. (2017). Aging Schwann cells: Mechanisms, implications, future directions. Curr. Opin. Neurobiol..

[31] Toews A.D., Barrett C., Morell P. (1998). Monocyte chemoattractant protein 1 is responsible for macrophage recruitment following injury to sciatic nerve. J. Neurosci. Res..

[32] Turner B.J., Ackerley S., Davies K.E., Talbot K. (2010). Dismutase-competent SOD1 mutant accumulation in myelinating Schwann cells is not detrimental to normal or transgenic ALS model mice. Hum. Mol. Genet..

[33] Lobsiger C.S., Boillee S., McAlonis-Downes M., Khan A.M., Feltri M.L., Yamanaka K., Cleveland D.W. (2009). Schwann cells expressing dismutase active mutant SOD1 unexpectedly slow disease progression in ALS mice. Proc. Natl. Acad. Sci. USA.

[34] Wang L., Pytel P., Feltri M.L., Wrabetz L., Roos R.P. (2012). Selective knockdown of mutant SOD1 in Schwann cells ameliorates disease in G85R mutant SOD1 transgenic mice. Neurobiol. Dis..

[35] Arbour D., Vande Velde C., Robitaille R. (2017). New perspectives on amyotrophic lateral sclerosis: The role of glial cells at the neuromuscular junction. J. Physiol..

[36] Carrasco D.I., Seburn K.L., Pinter M.J. (2016). Altered terminal Schwann cell morphology precedes denervation in SOD1 mice. Exp. Neurol..

[37] Carrasco D.I., Bahr B.A., Seburn K.L., Pinter M.J. (2016). Abnormal response of distal Schwann cells to denervation in a mouse model of motor neuron disease. Exp. Neurol..

[38] Trias E., Ibarburu S., Barreto-Nunez R., Varela V., Moura I.C., Dubreuil P., Hermine O., Beckman J.S., Barbeito L. (2017). Evidence for mast cells contributing to neuromuscular pathology in an inherited model of ALS. JCI Insight.

[39] Trias E., Ibarburu S., Barreto-Nunez R., Babdor J., Maciel T.T., Guillo M., Gros L., Dubreuil P., Diaz-Amarilla P., Cassina P. (2016). Post-paralysis tyrosine kinase inhibition with masitinib abrogates neuroinflammation and slows disease progression in inherited amyotrophic lateral sclerosis. J. Neuroinflamm..

[40] Hartlehnert M., Derksen A., Hagenacker T., Kindermann D., Schafers M., Pawlak M., Kieseier B.C., Meyer Zu Horste G. (2017). Schwann cells promote post-traumatic nerve inflammation and neuropathic pain through MHC class Ii. Sci. Rep..

[41] Burden S.J., Yumoto N., Zhang W. (2013). The role of MuSK in synapse formation and neuromuscular disease. Cold Spring Harb. Perspect. Biol..

[42] Marcuzzo S., Zucca I., Mastropietro A., de Rosbo N.K., Cavalcante P., Tartari S., Bonanno S., Preite L., Mantegazza R., Bernasconi P. (2011). Hind limb muscle atrophy precedes cerebral neuronal degeneration in G93A-SOD1 mouse model of amyotrophic lateral sclerosis: A longitudinal MRI study. Exp. Neurol..

[43] Hegedus J., Putman C.T., Gordon T. (2007). Time course of preferential motor unit loss in the SOD1 G93A mouse model of amyotrophic lateral sclerosis. Neurobiol. Dis..

[44] Dengler R., Konstanzer A., Kuther G., Hesse S., Wolf W., Struppler A. (1990). Amyotrophic lateral sclerosis: Macro-EMG and twitch forces of single motor units. Muscle Nerve.

[45] Sanjak M., Brinkmann J., Belden D.S., Roelke K., Waclawik A., Neville H.E., Ringel S.P., Murphy J.R., Brooks B.R. (2001). Quantitative assessment of motor fatigue in amyotrophic lateral sclerosis. J. Neurol. Sci..

[46] Wong M., Martin L.J. (2010). Skeletal muscle-restricted expression of human SOD1 causes motor neuron degeneration in transgenic mice. Hum. Mol. Genet..

[47] Dobrowolny G., Aucello M., Rizzuto E., Beccafico S., Mammucari C., Boncompagni S., Belia S., Wannenes F., Nicoletti C., Del Prete Z. (2008). Skeletal muscle is a primary target of SOD1^G93A^-mediated toxicity. Cell Metab..

[48] Garlepp M.J., Chen W., Tabarias H., Baines M., Brooks A., McCluskey J. (1995). Antigen processing and presentation by a murine myoblast cell line. Clin. Exp. Immunol..

[49] Wiendl H., Mitsdoerffer M., Schneider D., Melms A., Lochmuller H., Hohlfeld R., Weller M. (2003). Muscle fibres and cultured muscle cells express the B7.1/2-related inducible co-stimulatory molecule, ICOSL: Implications for the pathogenesis of inflammatory myopathies. Brain.

[50] Das L., Blumbergs P.C., Manavis J., Limaye V.S. (2013). Major histocompatibility complex class I and II expression in idiopathic inflammatory myopathy. Appl. Immunohistochem. Mol. Morphol..

[51] Englund P., Lindroos E., Nennesmo I., Klareskog L., Lundberg I.E. (2001). Skeletal muscle fibers express major histocompatibility complex class II antigens independently of inflammatory infiltrates in inflammatory myopathies. Am. J. Pathol..

[52] Wiendl H., Behrens L., Maier S., Johnson M.A., Weiss E.H., Hohlfeld R. (2000). Muscle fibers in inflammatory myopathies and cultured myoblasts express the nonclassical major histocompatibility antigen HLA-G. Ann. Neurol..

[53] Wiendl H., Mitsdoerffer M., Weller M. (2003). Express and protect yourself: The potential role of HLA-G on muscle cells and in inflammatory myopathies. Hum. Immunol..

[54] Rouas-Freiss N., Goncalves R.M., Menier C., Dausset J., Carosella E.D. (1997). Direct evidence to support the role of HLA-G in protecting the fetus from maternal uterine natural killer cytolysis. Proc. Natl. Acad. Sci. USA.

[55] Riteau B., Rouas-Freiss N., Menier C., Paul P., Dausset J., Carosella E.D. (2001). HLA-G2, -G3, and -G4 isoforms expressed as nonmature cell surface glycoproteins inhibit NK and antigen-specific CTl cytolysis. J. Immunol..

[56] Wiendl H., Mitsdoerffer M., Schneider D., Chen L., Lochmuller H., Melms A., Weller M. (2003). Human muscle cells express a B7-related molecule, B7-H1, with strong negative immune regulatory potential: A novel mechanism of counterbalancing the immune attack in idiopathic inflammatory myopathies. FASEB J..

[57] Waschbisch A., Wintterle S., Lochmuller H., Walter M.C., Wischhusen J., Kieseier B.C., Wiendl H. (2008). Human muscle cells express the costimulatory molecule B7-H3, which modulates muscle-immune interactions. Arthritis Rheum..

[58] Kawasaki T., Kawai T. (2014). Toll-like receptor signaling pathways. Front. Immunol..

[59] Zhao W., Beers D.R., Henkel J.S., Zhang W., Urushitani M., Julien J.P., Appel S.H. (2010). Extracellular mutant SOD1 induces microglial-mediated motoneuron injury. Glia.

[60] Nguyen M.D., D’Aigle T., Gowing G., Julien J.P., Rivest S. (2004). Exacerbation of motor neuron disease by chronic stimulation of innate immunity in a mouse model of amyotrophic lateral sclerosis. J. Neurosci..

[61] Kang J., Rivest S. (2007). MyD88-deficient bone marrow cells accelerate onset and reduce survival in a mouse model of amyotrophic lateral sclerosis. J. Cell Biol..

[62] Warren G.L., Hulderman T., Liston A., Simeonova P.P. (2011). Toll-like and adenosine receptor expression in injured skeletal muscle. Muscle Nerve.

[63] Schreiner B., Voss J., Wischhusen J., Dombrowski Y., Steinle A., Lochmuller H., Dalakas M., Melms A., Wiendl H. (2006). Expression of toll-like receptors by human muscle cells in vitro and in vivo: TLR3 is highly expressed in inflammatory and HIV myopathies, mediates IL-8 release and up-regulation of NKG2D-ligands. FASEB J..

[64] Pillon N.J., Krook A. (2017). Innate immune receptors in skeletal muscle metabolism. Exp. Cell Res..

[65] Henriques-Pons A., Yu Q., Rayavarapu S., Cohen T.V., Ampong B., Cha H.J., Jahnke V., Van der Meulen J., Wang D., Jiang W. (2014). Role of toll-like receptors in the pathogenesis of dystrophin-deficient skeletal and heart muscle. Hum. Mol. Genet..

[66] Fellner A., Barhum Y., Angel A., Perets N., Steiner I., Offen D., Lev N. (2017). Toll-like receptor-4 inhibitor TAK-242 attenuates motor dysfunction and spinal cord pathology in an amyotrophic lateral sclerosis mouse model. Int. J. Mol. Sci..

[67] Letiembre M., Liu Y., Walter S., Hao W., Pfander T., Wrede A., Schulz-Schaeffer W., Fassbender K. (2009). Screening of innate immune receptors in neurodegenerative diseases: A similar pattern. Neurobiol. Aging.

[68] Sta M., Sylva-Steenland R.M., Casula M., de Jong J.M., Troost D., Aronica E., Baas F. (2011). Innate and adaptive immunity in amyotrophic lateral sclerosis: Evidence of complement activation. Neurobiol. Dis..

[69] Ferraiuolo L., Heath P.R., Holden H., Kasher P., Kirby J., Shaw P.J. (2007). Microarray analysis of the cellular pathways involved in the adaptation to and progression of motor neuron injury in the SOD1 G93A mouse model of familial ALS. J. Neurosci..

[70] Lobsiger C.S., Boillee S., Cleveland D.W. (2007). Toxicity from different SOD1 mutants dysregulates the complement system and the neuronal regenerative response in ALS motor neurons. Proc. Natl. Acad. Sci. USA.

[71] Woodruff T.M., Costantini K.J., Crane J.W., Atkin J.D., Monk P.N., Taylor S.M., Noakes P.G. (2008). The complement factor C5a contributes to pathology in a rat model of amyotrophic lateral sclerosis. J. Immunol..

[72] Heurich B., El Idrissi N.B., Donev R.M., Petri S., Claus P., Neal J., Morgan B.P., Ramaglia V. (2011). Complement upregulation and activation on motor neurons and neuromuscular junction in the SOD1 G93A mouse model of familial amyotrophic lateral sclerosis. J. Neuroimmunol..

[73] Bahia El Idrissi N., Bosch S., Ramaglia V., Aronica E., Baas F., Troost D. (2016). Complement activation at the motor end-plates in amyotrophic lateral sclerosis. J. Neuroinflamm..

[74] Presumey J., Bialas A.R., Carroll M.C. (2017). Complement system in neural synapse elimination in development and disease. Adv. Immunol..

[75] Wang H.A., Lee J.D., Lee K.M., Woodruff T.M., Noakes P.G. (2017). Complement C5a-C5aR1 signalling drives skeletal muscle macrophage recruitment in the hSOD1 G93A mouse model of amyotrophic lateral sclerosis. Skelet. Muscle.

[76] Figarella-Branger D., Civatte M., Bartoli C., Pellissier J.F. (2003). Cytokines, chemokines, and cell adhesion molecules in inflammatory myopathies. Muscle Nerve.

[77] Jain A., Sharma M.C., Sarkar C., Bhatia R., Singh S., Handa R. (2009). Increased expression of cell adhesion molecules in inflammatory myopathies: Diagnostic utility and pathogenetic insights. Folia Neuropathol..

[78] Peake J.M., Della Gatta P., Suzuki K., Nieman D.C. (2015). Cytokine expression and secretion by skeletal muscle cells: Regulatory mechanisms and exercise effects. Exerc. Immunol. Rev..

[79] Tidball J.G. (2017). Regulation of muscle growth and regeneration by the immune system. Nat. Rev. Immunol..

[80] Heredia J.E., Mukundan L., Chen F.M., Mueller A.A., Deo R.C., Locksley R.M., Rando T.A., Chawla A. (2013). Type 2 innate signals stimulate fibro/adipogenic progenitors to facilitate muscle regeneration. Cell.

[81] Liu G.T., Hwang C.S., Hsieh C.H., Lu C.H., Chang S.L., Lee J.C., Huang C.F., Chang H.T. (2013). Eosinophil-derived neurotoxin is elevated in patients with amyotrophic lateral sclerosis. Mediat. Inflamm..

[82] Rentzos M., Nikolaou C., Rombos A., Boufidou F., Zoga M., Dimitrakopoulos A., Tsoutsou A., Vassilopoulos D. (2007). Rantes levels are elevated in serum and cerebrospinal fluid in patients with amyotrophic lateral sclerosis. Amyotroph. Lateral Scler..

[83] Schroder T., Fuchss J., Schneider I., Stoltenburg-Didinger G., Hanisch F. (2013). Eosinophils in hereditary and inflammatory myopathies. Acta Myol..

[84] Sunohara N., Furukawa S., Nishio T., Mukoyama M., Satoyoshi E. (1989). Neurotoxicity of human eosinophils towards peripheral nerves. J. Neurol. Sci..

[85] Kingham P.J., McLean W.G., Walsh M.T., Fryer A.D., Gleich G.J., Costello R.W. (2003). Effects of eosinophils on nerve cell morphology and development: The role of reactive oxygen species and p38 MAP kinase. Am. J. Physiol. Lung Cell. Mol. Physiol..

[86] Walsh M.T., Curran D.R., Kingham P.J., Morgan R.K., Durcan N., Gleich G.J., McLean W.G., Costello R.W. (2004). Effect of eosinophil adhesion on intracellular signaling in cholinergic nerve cells. Am. J. Respir. Cell Mol. Biol..

[87] Yu C., Cantor A.B., Yang H., Browne C., Wells R.A., Fujiwara Y., Orkin S.H. (2002). Targeted deletion of a high-affinity GATA-binding site in the GATA-1 promoter leads to selective loss of the eosinophil lineage in vivo. J. Exp. Med..

[88] Burzyn D., Kuswanto W., Kolodin D., Shadrach J.L., Cerletti M., Jang Y., Sefik E., Tan T.G., Wagers A.J., Benoist C. (2013). A special population of regulatory T cells potentiates muscle repair. Cell.

[89] Kuswanto W., Burzyn D., Panduro M., Wang K.K., Jang Y.C., Wagers A.J., Benoist C., Mathis D. (2016). Poor repair of skeletal muscle in aging mice reflects a defect in local, interleukin-33-dependent accumulation of regulatory T cells. Immunity.

[90] Lin C.Y., Pfluger C.M., Henderson R.D., McCombe P.A. (2012). Reduced levels of interleukin 33 and increased levels of soluble ST2 in subjects with amyotrophic lateral sclerosis. J. Neuroimmunol..

[91] Gadani S.P., Walsh J.T., Smirnov I., Zheng J., Kipnis J. (2015). The glia-derived alarmin IL-33 orchestrates the immune response and promotes recovery following CNS injury. Neuron.

[92] Chapuis J., Hot D., Hansmannel F., Kerdraon O., Ferreira S., Hubans C., Maurage C.A., Huot L., Bensemain F., Laumet G. (2009). Transcriptomic and genetic studies identify IL-33 as a candidate gene for alzheimer’s disease. Mol. Psychiatry.

[93] Jiang H.R., Milovanovic M., Allan D., Niedbala W., Besnard A.G., Fukada S.Y., Alves-Filho J.C., Togbe D., Goodyear C.S., Linington C. (2012). IL-33 attenuates eae by suppressing IL-17 and IFN-γ production and inducing alternatively activated macrophages. Eur. J. Immunol..

[94] Huang P.L., Hou M.S., Wang S.W., Chang C.L., Liou Y.H., Liao N.S. (2015). Skeletal muscle interleukin 15 promotes CD8^+^ T-cell function and autoimmune myositis. Skelet. Muscle.

[95] Lutz C.T., Quinn L.S. (2012). Sarcopenia, obesity, and natural killer cell immune senescence in aging: Altered cytokine levels as a common mechanism. Aging.

[96] Rentzos M., Rombos A., Nikolaou C., Zoga M., Zouvelou V., Dimitrakopoulos A., Alexakis T., Tsoutsou A., Samakovli A., Michalopoulou M. (2010). Interleukin-15 and interleukin-12 are elevated in serum and cerebrospinal fluid of patients with amyotrophic lateral sclerosis. Eur. Neurol..

[97] Gustafson M.P., Staff N.P., Bornschlegl S., Butler G.W., Maas M.L., Kazamel M., Zubair A., Gastineau D.A., Windebank A.J., Dietz A.B. (2017). Comprehensive immune profiling reveals substantial immune system alterations in a subset of patients with amyotrophic lateral sclerosis. PLoS ONE.

[98] Valdez G., Tapia J.C., Lichtman J.W., Fox M.A., Sanes J.R. (2012). Shared resistance to aging and ALS in neuromuscular junctions of specific muscles. PLoS ONE.

[99] Holtman I.R., Raj D.D., Miller J.A., Schaafsma W., Yin Z., Brouwer N., Wes P.D., Moller T., Orre M., Kamphuis W. (2015). Induction of a common microglia gene expression signature by aging and neurodegenerative conditions: A co-expression meta-analysis. Acta Neuropathol. Commun..

[100] Hammarberg H., Lidman O., Lundberg C., Eltayeb S.Y., Gielen A.W., Muhallab S., Svenningsson A., Linda H., van Der Meide P.H., Cullheim S. (2000). Neuroprotection by encephalomyelitis: Rescue of mechanically injured neurons and neurotrophin production by CNS-infiltrating T and natural killer cells. J. Neurosci..

[101] Gross C.C., Schulte-Mecklenbeck A., Wiendl H., Marcenaro E., Kerlero de Rosbo N., Uccelli A., Laroni A. (2016). Regulatory functions of natural killer cells in multiple sclerosis. Front. Immunol..

[102] Jadidi-Niaragh F., Shegarfi H., Naddafi F., Mirshafiey A. (2012). The role of natural killer cells in Alzheimer’s disease. Scand. J. Immunol..

[103] Lincecum J.M., Vieira F.G., Wang M.Z., Thompson K., De Zutter G.S., Kidd J., Moreno A., Sanchez R., Carrion I.J., Levine B.A. (2010). From transcriptome analysis to therapeutic anti-CD40l treatment in the SOD1 model of amyotrophic lateral sclerosis. Nat. Genet..

[104] Pfohl S.R., Halicek M.T., Mitchell C.S. (2015). Characterization of the contribution of genetic background and gender to disease progression in the SOD1 G93A mouse model of amyotrophic lateral sclerosis: A meta-analysis. J. Neuromuscul. Dis..

[105] Murdock B.J., Bender D.E., Kashlan S.R., Figueroa-Romero C., Backus C., Callaghan B.C., Goutman S.A., Feldman E.L. (2016). Increased ratio of circulating neutrophils to monocytes in amyotrophic lateral sclerosis. Neurol. Neuroimmunol. Neuroinflamm..

[106] Rusconi M., Gerardi F., Santus W., Lizio A., Sansone V.A., Lunetta C., Zanoni I., Granucci F. (2017). Inflammatory role of dendritic cells in amyotrophic lateral sclerosis revealed by an analysis of patients’ peripheral blood. Sci. Rep..

[107] Henkel J.S., Beers D.R., Wen S., Rivera A.L., Toennis K.M., Appel J.E., Zhao W., Moore D.H., Powell S.Z., Appel S.H. (2013). Regulatory T-lymphocytes mediate amyotrophic lateral sclerosis progression and survival. EMBO Mol. Med..

[108] Cudkowicz M.E., Shefner J.M., Schoenfeld D.A., Zhang H., Andreasson K.I., Rothstein J.D., Drachman D.B. (2006). Trial of celecoxib in amyotrophic lateral sclerosis. Ann. Neurol..

[109] Calvo A., Moglia C., Balma M., Chio A. (2010). Involvement of immune response in the pathogenesis of amyotrophic lateral sclerosis: A therapeutic opportunity?. CNS Neurol. Disord. Drug Targets.

[110] Smith S.A., Miller R.G., Murphy J.R., Ringel S.P. (1994). Treatment of ALS with high dose pulse cyclophosphamide. J. Neurol. Sci..

[111] Werdelin L., Boysen G., Jensen T.S., Mogensen P. (1990). Immunosuppressive treatment of patients with amyotrophic lateral sclerosis. Acta Neurol. Scand..

[112] Crisafulli S.G., Brajkovic S., Cipolat Mis M.S., Parente V., Corti S. (2017). Therapeutic strategies under development targeting inflammatory mechanisms in amyotrophic lateral sclerosis. Mol. Neurobiol..

